# Precise diagnosis and management of non-traumatic radial nerve palsy attributed to multiple rare etiologies: case study and literature review

**DOI:** 10.3389/fmed.2026.1900239

**Published:** 2026-08-28

**Authors:** Yanchen Dong, Nanfei Zheng, Hongyue Men, Chen Xie

**Affiliations:** 1 Outpatient Department, The 960th Hospital of PLA, Jinan, Shandong, China; 2 Department of Hand Surgery, The 960th Hospital of PLA, Jinan, Shandong, China

**Keywords:** acupotomy therapy, hourglass-like constriction, iatrogenic injury, precise diagnosis and management, radial nerve palsy

## Abstract

While non-traumatic radial nerve palsy is relatively rare, cases triggered by a single etiological factor pose minimal diagnostic and therapeutic challenges. However, for radial nerve palsy driven by multiple factors, the overlapping or masking effects of symptoms and signs from distinct etiologies complicate accurate diagnosis and optimal management. This complexity is further amplified when all contributing factors are rare, presenting a more formidable challenge to precise diagnosis and treatment selection. We present a case of radial nerve palsy attributed to multiple etiological factors, which is notable for the coexistence of three distinct and rare pathogenic entities: elbow joint cyst, hourglass-like constriction, and a specific iatrogenic injury—a combination that is rarely, if ever, documented in the existing literature. Through the synthesis of clinical experience from the diagnostic and therapeutic process and a comprehensive literature review, it aims to provide valuable references for the precise diagnosis and management of such rare clinical entities.

## Introduction

The overall incidence of radial nerve palsy is approximately 4%−22% (1), with the vast majority linked to humeral shaft fractures. In contrast, lesions involving the distal branches of the radial nerve (e.g., the deep branch of the radial nerve) are relatively rare, with an incidence of only about 0.03% (2). Mechanical entrapment is the primary cause of deep radial nerve palsy, commonly attributed to factors such as hypertrophy of the supinator arch or masses around the elbow joint. However, a very small subset of cases lack external compression, instead presenting with spontaneous nerve narrowing that forms an hourglass-like constriction. This condition is termed neuralgic amyotrophy or Parsonage-Turner syndrome (3–5), an even rarer entity with only scattered case reports in the literature (6).

Acupotomy therapy, a form of acupuncture intervention, is frequently employed for the management of localized pain due to its minimally invasive nature and favorable efficacy. Nevertheless, in the absence of imaging-guided localization, there exists a risk of iatrogenic injury to deep critical structures. While a limited number of reports have documented tendon injuries associated with acupotomy, no cases of nerve injury resulting from this procedure have been previously reported.

In this article, we present a retrospective analysis of a patient with radial nerve palsy attributed to the three aforementioned rare etiologies. Satisfactory clinical outcomes were achieved following surgical intervention. By reviewing and analyzing the diagnostic and therapeutic trajectory of this case, this report aims to provide valuable insights for the precise diagnosis and management of patients with radial nerve palsy secondary to multiple rare causal factors.

## Case presentation

A 57-year-old female farmer was admitted to the hospital due to sudden-onset pain in the left hand and left forearm without obvious triggering factors 1 month before admission. 1 week after the onset of symptoms, she gradually developed motor dysfunction, manifesting as weakened finger and wrist extension strength. 2 weeks after the onset, she sought medical attention at a local clinic and received acupotomy therapy; however, no improvement in sensory and motor dysfunction was observed after the treatment.

**Figure 1:**
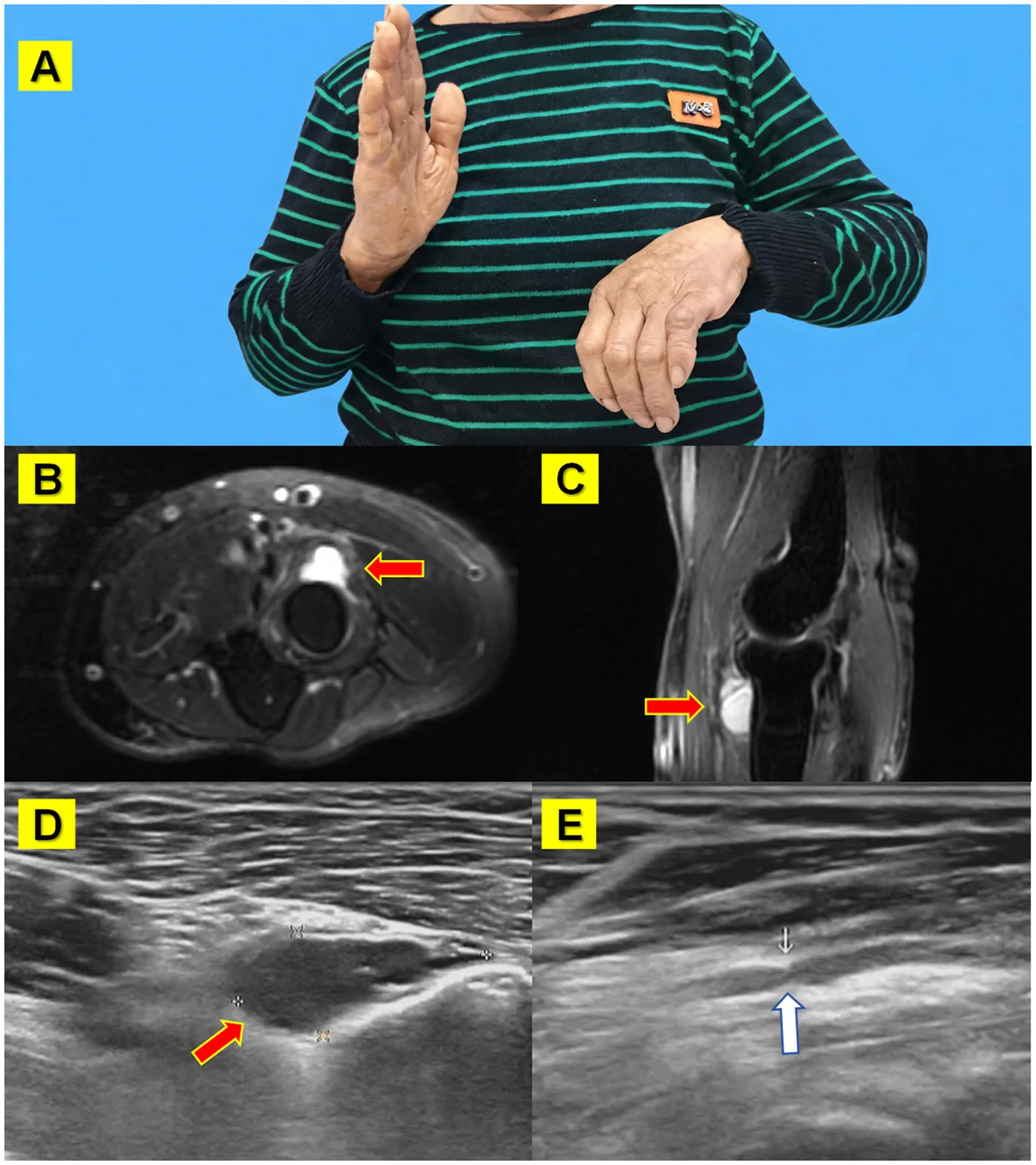
**(A)** Preoperative posture of the affected limb, demonstrating wrist drop deformity. **(B–C)** T2-weighted fat-suppressed axial and sagittal MRI images, show a high-signal cystic mass measuring approximately 2.5 cm × 1.5 cm, located in the superficial distal portion of the radial head and the deep proximal portion of the supinator muscle (red arrow). **(D)** Longitudinal ultrasound section revealed a cyst anterior to the elbow joint, measuring approximately 2.5 cm × 1.5 cm. (red arrow). **(E)** An hourglass-like constriction was identified at the proximal portion of the deep branch of the radial nerve (white arrow).

Admission Physical Examination Findings: the left upper limb exhibited a “wrist drop” deformity, with near-complete loss of active dorsiflexion of the wrist and fingers. The muscle strength of finger and thumb extension was Grade 0, wrist extension strength was approximately Grade 2, while flexion strength was within the normal range (Figure 1A). Sensory abnormalities were observed in the dorsal aspect of the hand and forearm. Needle electromyography (EMG) revealed markedly increased insertional activity, fibrillation potentials, and positive sharp waves, with no motor unit action potentials (MUAPs) detected. Motor nerve conduction studies failed to elicit compound muscle action potentials (CMAPs). Sensory nerve conduction showed mildly decreased amplitude and conduction velocity (Table 1). T2-weighted fat-saturated Magnetic Resonance Imaging (MRI) images show a high-signal cystic mass measuring approximately 2.5 cm × 1.5 cm, located in the superficial distal portion of the radial head and the deep proximal portion of the supinator muscle (Figures 1B, C). Ultrasound examination revealed that the cyst was located deep to the deep branch of the radial nerve, with corresponding attenuation of the deep branch at that site. In addition, an hourglass-like constriction was identified at the proximal portion of the deep branch of the radial nerve; the diameter at the constricted segment was approximately 1 mm, whereas that of the normal segments on either side was about 3 mm. The fascicular architecture at the constriction was disorganized, and no obvious blood flow was detected (Figures 1D, E).

**Table 1:** Electrophysiological examination results.

Parameter		Preop	3 mo postop	3 yr postop
EMG	IA	+	+	N/A
	Fib	+++	+++	N/A
	PSW	+++	++++	N/A
MNCS	Lat (ms)	NR	NR	1.3
	Amp (mV)	NR	NR	10.6
	CV (m/s)	NR	NR	45.4
SNCS	Lat (ms)	2.2	1.3	2.5
	Amp (mV)	10	16	18
	CV (m/s)	38.8	53.9	46.7

IA, insertional activity; Fib, fibrillation potentials; PSW, positive sharp waves; Lat, latency; Amp, amplitude; CV, conduction velocity; NR, no response; N/A, not available (patient declined needle EMG at 3-year follow-up). + indicates mild (persistent abnormal spontaneous activity in at least two different insertion sites);+++indicates severe (abundant spontaneous activity present in most examined areas);++++indicates very severe (extremely dense spontaneous activity, nearly filling the baseline, observed in all examined areas).

The patient had no history of trauma and presented with sudden-onset pain as the initial symptom, which subsequently progressed to gradually worsening weakness in wrist and finger extension. This clinical manifestation aligns with the diagnostic criteria for neuralgic amyotrophy, allowing for a preliminary localization of the lesion to the radial nerve. Subsequent imaging and electrophysiological investigations confirmed the initial clinical hypothesis and precisely delineated the anatomical site of the lesion. The patient had a one-month disease course, during which conservative management (including acupotomy therapy) was administered without substantial clinical improvement. Additionally, imaging studies revealed definitive external compression and nerve pathology, which warranted surgical intervention. Thus, we determined that the patient met the surgical indications and elected to perform radial nerve exploration.

**Figure 2:**
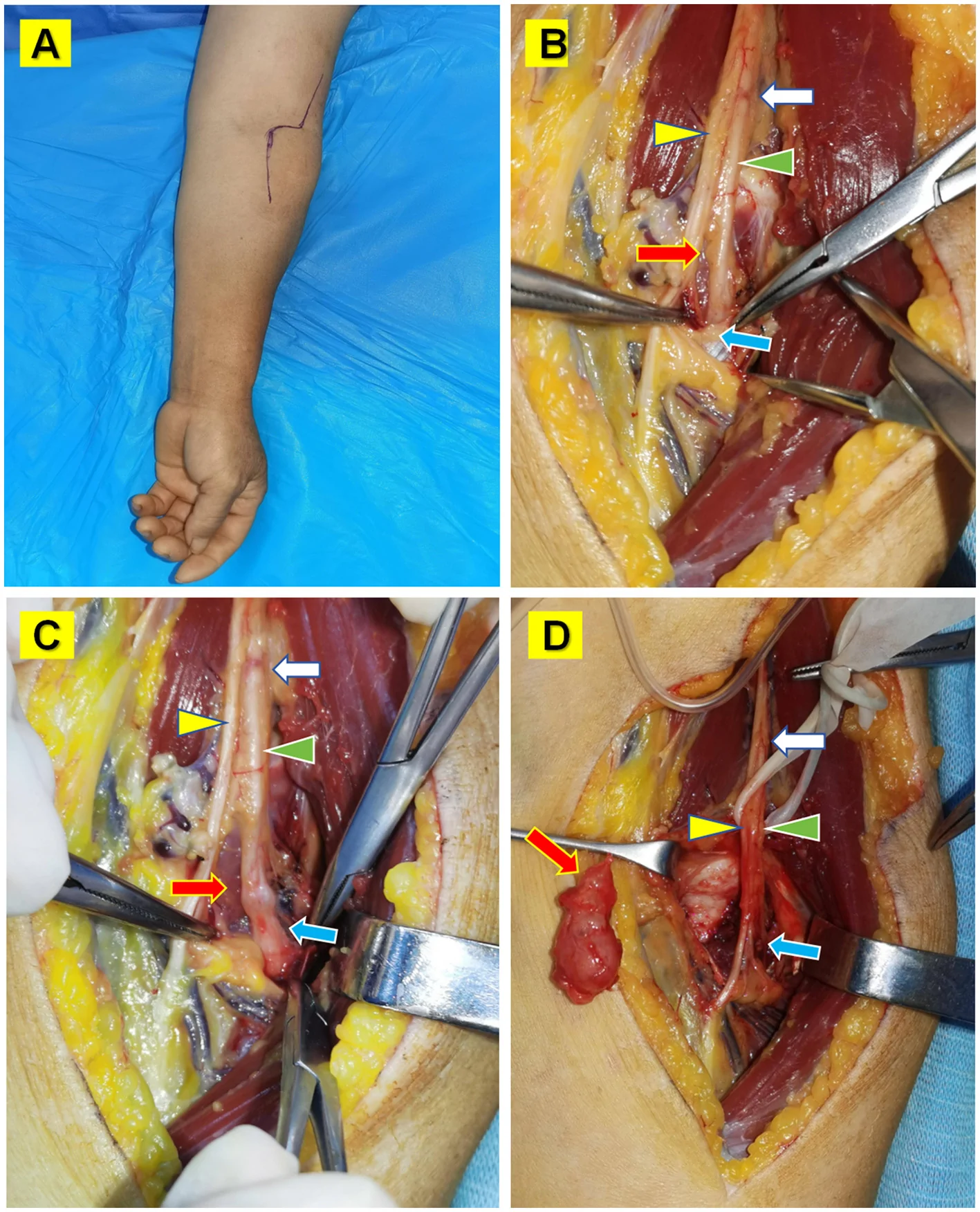
**(A)** Surgical incision. **(B)** Exploration and exposure of the deep branch (green triangle) and superficial branch (yellow triangle) of the radial nerve: the nerve was elevated and compressed by a deep-seated cyst (red arrow), leading to entrapment at the arcade of Frohse (blue arrow); additionally, a hourglass-like constriction was noted in the proximal segment of the deep branch (white arrow). **(C)** Following incision of the arcade of Frohse, the compressed nerve segment was found to be attenuated (blue arrow), with proximal and distal thickening. **(D)** Complete excision of the cyst (red arrow) and release of the hourglass-like constriction (white arrow).

Surgery was performed via an anterior elbow incision for radial nerve exploration (Figure 2A). The main trunk, superficial branch, and deep branch of the radial nerve were exposed. Intraoperative exploration revealed an hourglass-like constriction in the proximal segment of the deep branch of the radial nerve, approximately 2 cm to the bifurcation of the superficial and deep branches. At the entry point of the nerve into the supinator muscle, the nerve was elevated and compressed by a deep-seated cyst, leading to entrapment at the arcade of Frohse (supinator tendon arch) (Figure 2B). The compressed nerve segment exhibited tapering, while the nerve segments proximal and distal to the compression site were enlarged (Figure 2C).

Intraoperatively, the arcade of Frohse was incised, followed by complete mobilization and decompression of the deep branch of the radial nerve, which was then retracted for protection. The deep-seated cyst was completely excised, with dimensions measuring approximately 2.5 cm × 1.5 cm. Subsequent exploration of the hourglass-like constriction site revealed localized tapering of the nerve, with a small number of intact nerve fascicles remaining contiguous. Given the preservation of partial normal nerve fibers at the lesion site, the affected nerve segment was retained, and epineural as well as perineurial release was performed on the lesion (Figure 2D).

Postoperatively, the affected limb was immobilized with a brace for 6 weeks, and neurotrophic agents were administered continuously. At the 6-week postoperative follow-up, the brace was removed; sensory abnormalities showed partial improvement, while motor function remained unrecovered. At the 3-month postoperative re-evaluation, sensory disturbances had essentially resolved with no pain or numbness reported. However, motor function still showed no signs of recovery, and the electromyography and nerve conduction study findings also showed no significant improvement compared with the preoperative results (Table 1).

We analyzed the potential etiologies as follows: first, complete cyst resection was achieved during the initial surgery, theoretically alleviating the associated compression; however, the recovery status of the lesion at the hourglass-like constriction remains undetermined. Second, there may be undiagnosed underlying pathogenic factors.

Upon further in-depth medical history inquiry with the patient and their family, a previously unrecognized detail was identified: the family reported that before acupotomy therapy, the patient had significantly reduced wrist and finger extension strength but could still achieve minimal elevation; however, motor function became completely absent immediately after the procedure. The abrupt exacerbation of motor dysfunction following the intervention raises suspicion of potential iatrogenic injury, despite the extremely low probability of nerve damage caused by the fine needle.

**Figure 3:**
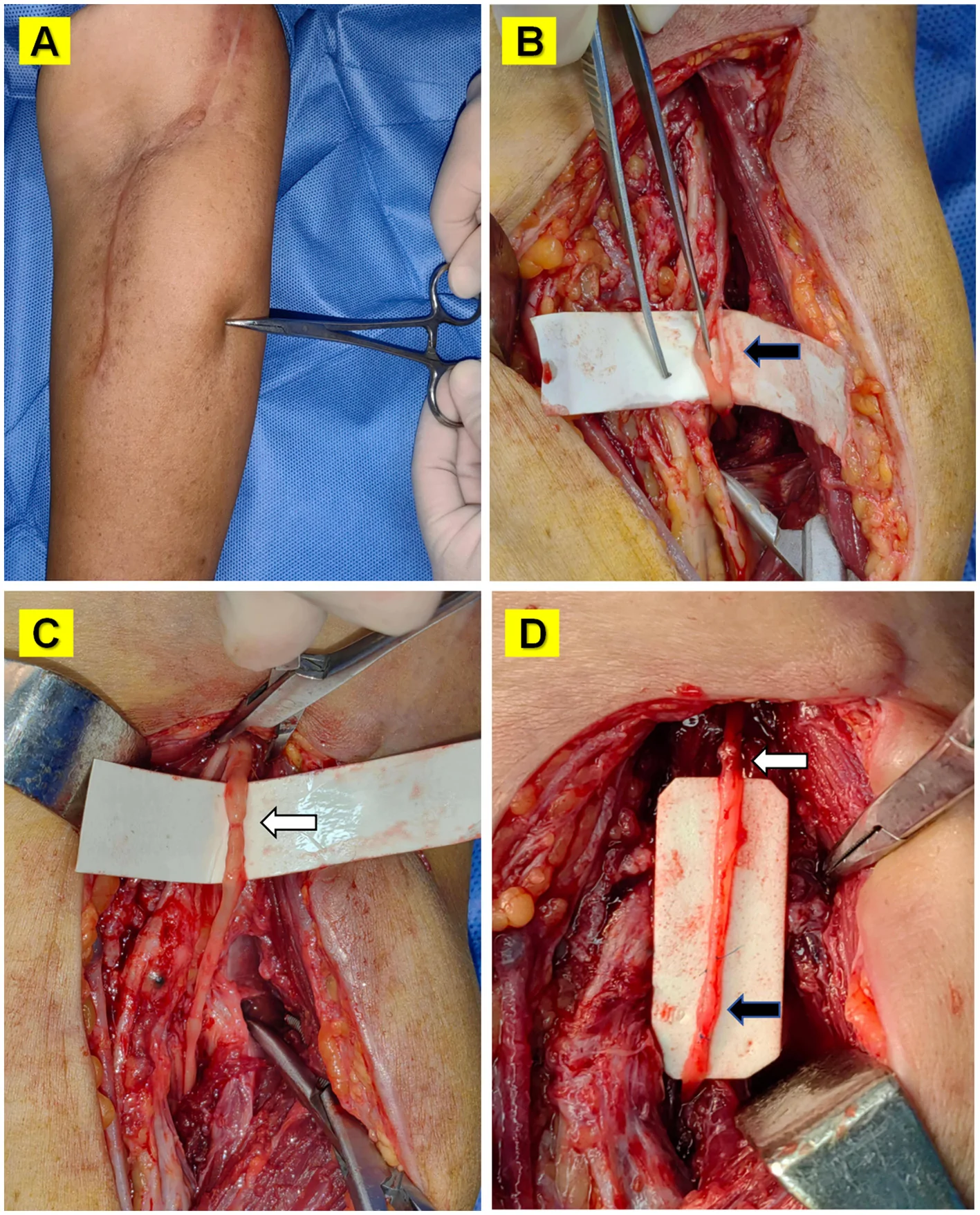
**(A)** The hemostatic forceps indicate the needle knife insertion site as described by the patient. **(B)** After incising the supinator muscle and exploring distally, the deep branch of the radial nerve was found to be lacerated deep within the supinator muscle, forming a rupture (black arrow). **(C)** The hourglass-like constriction (white arrow) that was released during the previous surgery showed no improvement. **(D)** The lacerated nerve was anastomosed (black arrow), followed by excision of the hourglass-like constriction segment and subsequent end-to-end neurorrhaphy (white arrow).

Following written informed consent from the patient and their legal next of kin, a repeat nerve exploration was performed (Figure 3A). Intraoperatively, the supinator muscle was incised distally, followed by exploration of the intrasupinator and more distal segments of the nerve. Eventually, a roughly 1-cm-long laceration was identified in the intrasupinator segment of the deep branch of the radial nerve (Figure 3B), which was considered an iatrogenic injury caused by the acupotomy. Additionally, we re-evaluated the previously decompressed nerve site: the nerve at the cyst compression location had fully normalized, while no improvement was observed at the hourglass-like constriction site (Figure 3C).

Accordingly, we performed debridement of the injured nerve at the laceration site, excised the superficial scar tissue, and, after exposing the normal nerve tissue, anastomosed the transected nerve fascicles. Subsequently, re-exploration and decompression were conducted at the hourglass-like constriction; it was found that almost no intact nerve fascicles were passing through the stenotic segment. Therefore, the nerve segment affected by the stenotic lesion was resected, and the proximal and distal stumps were directly anastomosed (Figure 3D).

**Figure 4:**
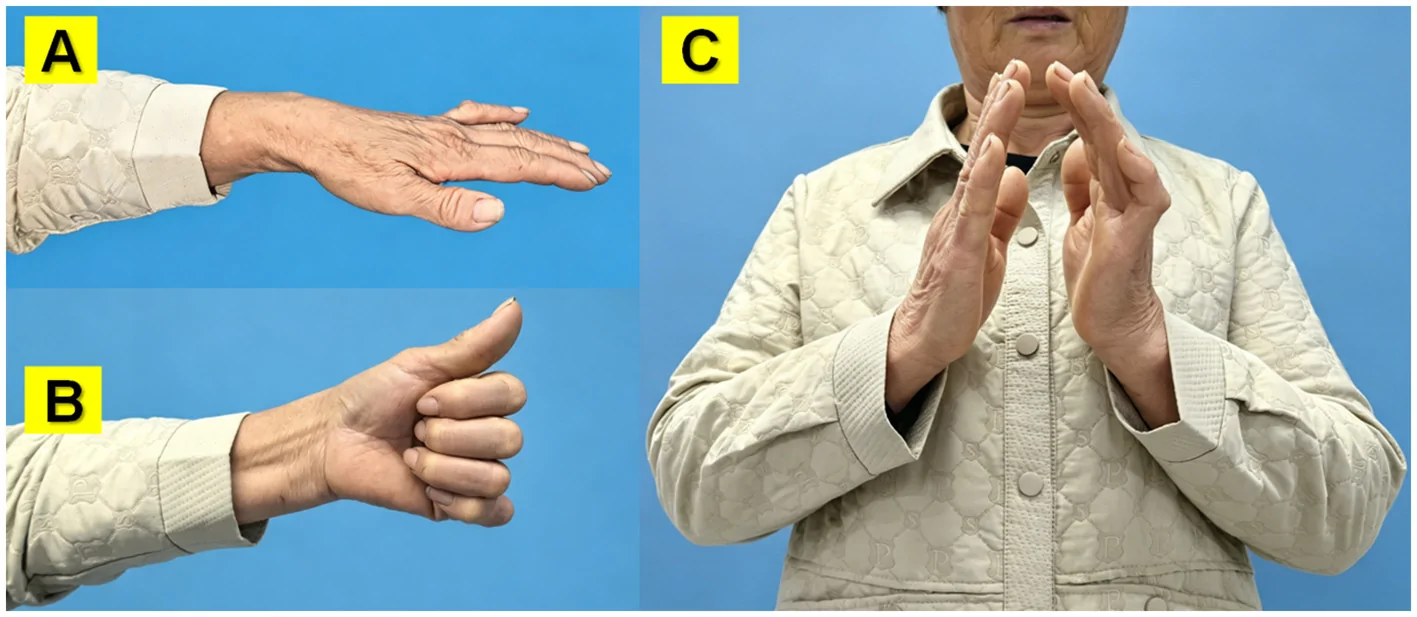
Three-year postoperative follow-up findings: **(A)** Wrist and finger extension muscle strength was approximately Grade 4. **(B)** Thumb extension muscle strength was approximately Grade 3. **(C)** The function of the affected limb was largely restored.

Postoperatively, the patient was primarily followed up via telephone. At the one-year postoperative follow-up, the patient self-reported mild improvement in motor function, although the effect was not significant. The patient was subsequently lost to follow-up for a period of time. At the three-year postoperative mark, we re-established contact with the patient and conducted an in-home follow-up visit. Physical examination revealed significant motor function recovery: muscle strength for wrist extension and finger extension reached Grade 4, while thumb extension strength was slightly weaker at approximately Grade 3; sensory function had returned to normal (Figure 4). Since the patient declined to undergo needle electromyography because of discomfort, we performed only nerve conduction studies at follow-up. The results showed that compound muscle action potentials could be successfully elicited and recorded, and the motor nerve conduction velocity, amplitude, and latency were all close to the normal range (Table 1).

## Discussion

Compression of the deep branch of the radial nerve, hourglass-like constriction, and iatrogenic injury induced by acupotomy therapy are all relatively rare or extremely rare etiologies of non-traumatic radial nerve palsy. The co-occurrence of these three distinct and uncommon causal factors within a single nerve branch is even more exceptional. This poses a substantial challenge to the accurate identification of the underlying pathological causes and the formulation of optimal therapeutic strategies.

Non-traumatic radial nerve palsy is predominantly attributed to mechanical compression. However, compared to other entrapment neuropathies (e.g., carpal tunnel syndrome and cubital tunnel syndrome), radial nerve entrapment disorders are relatively rare (7, 8). The most common compression sites include the peri-elbow region and the arcade of Frohse. The primary etiological factor for compression is hypertrophy of the arcade of Frohse, while additional causes involve compression by peri-elbow tumors or cysts (9–12). Since compression typically occurs distal to the branching point of the superficial radial nerve, the clinical manifestations are predominantly motor dysfunction, though occasional local pain may also be present (13). This bears a high degree of similarity to the clinical signs and symptoms of neuralgic amyotrophy induced by hourglass-like constriction. While the quality and location of pain may vary, differentiation based solely on clinical signs and symptoms remains challenging. Ultrasonography serves as the primary diagnostic modality, as it can accurately localize the compression site and assess the extent of nerve compromise, thereby offering critical guidance for therapeutic decision-making.

Hourglass-like constriction is a rare etiology of nerve palsy, characterized by spontaneous hourglass-like constriction of the nerve at the lesion site (14, 15). Its exact pathogenesis remains inconclusive, though it is primarily hypothesized to be associated with persistent inflammatory torsion of the nerve and genetic factors (6, 16). The cardinal symptoms include sudden-onset pain in the affected limb, followed by progressive weakness or even paralysis of muscles innervated by the involved nerve (6, 16, 17). A study has demonstrated that 33.5% of patients develop muscle paralysis within 24 h, 39.3% within the first week after pain onset, and a small subset present with delayed onset (18). Therefore, in cases of sudden onset of pain without identifiable precipitating factors accompanied by muscle weakness, the possibility of hourglass-like constriction should be considered. Historically, the diagnostic accuracy for hourglass-like constriction was relatively low. However, with advancements in ultrasound technology—particularly high-frequency ultrasound—the diagnostic precision for this condition has improved markedly. High-frequency ultrasound not only facilitates the detection of pathological lesions but also enables assessment of stenosis severity, thereby furnishing critical evidence to inform clinical therapeutic decision-making (19, 20). Therapeutic strategies for hourglass-like constriction encompass conservative management, surgical decompression, resection with anastomosis, or nerve reconstruction; however, the optimal treatment selection remains a subject of debate (21). Currently, most studies advocate for a comprehensive evaluation based on the severity of constriction, the integrity of the affected nerve, and the findings of intraoperative neurofunctional testing. For mild to moderate constriction, neurolysis alone is generally recommended as the preferred intervention; in cases of severe constriction or when tension at the suture site is anticipated, resection combined with nerve grafting may be considered (6, 17, 22). However, there remains a lack of precise quantitative criteria for evaluating the severity of constriction, and clinical decision-making in this regard still heavily relies on the surgeon's accumulated clinical experience.

Acupotomy therapy is a modality of acupuncture that employs a fine needle with a flat, blade-shaped tip (23). The therapeutic mechanism of acupotomy therapy involves using the flat blade at the needle tip to dissect and release contractures and adhesions in deep soft tissues, thereby achieving therapeutic outcomes (23, 24). Currently, it has been widely applied in the management of pain and certain compressive disorders, including cervical spondylosis, adhesive capsulitis of the shoulder, knee osteoarthritis, lumbar spinal stenosis, stenosing tenosynovitis, and carpal tunnel syndrome (25–29). However, there is a lack of systematic literature reporting on its safety profile and adverse events (24). To date, no literature has documented the application of acupotomy therapy for radial nerve compression disorders. In the present case, the primary indication for early-stage acupotomy therapy was likely forearm pain. McManus and Cleary (30) previously reported a case where dry needling for forearm pain resulted in radial nerve dysfunction; however, only conservative management was administered, leading to suboptimal recovery of nerve function.

In this case review, the patient presented with upper limb pain as the initial symptom. The community physician at the first visit, likely due to insufficient understanding of neurological disorders, was misled into administering an inadequately appropriate acupotomy therapy, which unfortunately caused iatrogenic nerve injury. As the disease progressed, cyst compression, hourglass-like constriction, and nerve injury all presented with paralysis of the forearm extensor muscle group; overlapping symptoms and signs mutually masked each other, further increasing the diagnostic difficulty. While high-precision imaging modalities, including high-frequency ultrasound (HFUS) and magnetic resonance imaging (MRI), are assuming an increasingly pivotal role in the diagnosis of peripheral nerve disorders (31–33), effectively improving diagnostic efficiency, they still depend on the examiner's understanding and knowledge of clinical conditions. In this case, imaging examinations identified the cyst and hourglass-like constriction but failed to detect the distal nerve injury. This may be attributed to two factors: from an objective standpoint, the radial nerve becomes considerably thinner after entering the supinator muscle, and the rupture was located in the middle portion of the nerve without causing complete transection. During examination, if the ultrasound probe was not aligned perpendicular to the plane of the rupture but rather approached from an oblique angle, the defect might have been missed. From a subjective perspective, the accuracy of ultrasonography is highly dependent on the examiner's experience and technical skill. An inexperienced examiner could easily overlook such an inconspicuous injury. Moreover, even for an experienced examiner, after identifying the two preceding distinct lesions, there might be a subconscious relaxation of vigilance or a tendency toward complacency, thereby missing the less obvious finding. As clinicians, on the one hand, we exhibited an overreliance on imaging findings, presuming that the two lesions identified by imaging were sufficient to account for the patient's clinical presentation without conducting a more meticulous exploration of the medical history. On the other hand, our insufficient awareness of the coexistence of multiple rare etiological factors contributed to the failure to establish an accurate and comprehensive diagnosis before the initial surgery.

Based on the foregoing literature review and retrospective case analysis, several evidence-informed clinical takeaways can be derived. Advanced imaging and molecular diagnostics significantly enhance diagnostic precision; however, overreliance on these technologies risks overlooking critical clinical clues. Accurate diagnosis continues to rest fundamentally on comprehensive history-taking, vigilant scrutiny of longitudinal disease evolution, and hypothesis-driven clinical reasoning. Acupotomy therapy, though minimally invasive and anatomically precise, demands rigorous patient selection, in-depth knowledge of regional neurovascular anatomy, and—ideally—real-time ultrasound guidance to ensure procedural safety and efficacy. Regarding the treatment of hourglass-like constriction, although mild constriction may achieve satisfactory outcomes through neurolysis alone, when most of the nerve fascicles in the lesioned area have been involved, even if a small number of normal nerve fibers remain, simple neurolysis is likely to be insufficient (as was demonstrated in the initial surgical treatment of this case). In such circumstances, complete resection of the lesion followed by nerve anastomosis or grafting may be a better choice, because this approach has generally yielded satisfactory outcomes, as supported by both the literature and our clinical practice.

This study has certain limitations. As it primarily involves a retrospective analysis and experience summary of a rare case, its conclusions incorporate some subjective components from the authors and thus cannot be regarded as high-level evidence-based medical evidence. The significance of this study lies primarily in providing a reference for clinicians when encountering similar rare cases, thereby reducing the risk of missed diagnoses or misdiagnosis and enabling patients to receive a more precise diagnosis and treatment.

## Data Availability

The original contributions presented in the study are included in the article/supplementary material, further inquiries can be directed to the corresponding author.
